# Size-Dependent Antimicrobial Effects of Novel Palladium Nanoparticles

**DOI:** 10.1371/journal.pone.0085981

**Published:** 2014-01-20

**Authors:** Clara P. Adams, Katherine A. Walker, Sherine O. Obare, Kathryn M. Docherty

**Affiliations:** 1 Department of Chemistry, Western Michigan University, Kalamazoo, Michigan, United States of America; 2 Department of Biological Sciences, Western Michigan University, Michigan, United States of America; Centro Nacional de Biotecnologia - CSIC, Spain

## Abstract

Investigating the interactions between nanoscale materials and microorganisms is crucial to provide a comprehensive, proactive understanding of nanomaterial toxicity and explore the potential for novel applications. It is well known that nanomaterial behavior is governed by the size and composition of the particles, though the effects of small differences in size toward biological cells have not been well investigated. Palladium nanoparticles (Pd NPs) have gained significant interest as catalysts for important carbon-carbon and carbon-heteroatom reactions and are increasingly used in the chemical industry, however, few other applications of Pd NPs have been investigated. In the present study, we examined the antimicrobial capacity of Pd NPs, which provides both an indication of their usefulness as target antimicrobial compounds, as well as their potency as potential environmental pollutants. We synthesized Pd NPs of three different well-constrained sizes, 2.0±0.1 nm, 2.5±0.2 nm and 3.1±0.2 nm. We examined the inhibitory effects of the Pd NPs and Pd^2+^ ions toward gram negative *Escherichia coli* (*E. coli*) and gram positive *Staphylococcus aureus* (*S. aureus*) bacterial cultures throughout a 24 hour period. Inhibitory growth effects of six concentrations of Pd NPs and Pd^2+^ ions (2.5×10^−4^, 10^−5^, 10^−6^, 10^−7^, 10^−8^, and 10^−9^ M) were examined. Our results indicate that Pd NPs are generally much more inhibitory toward *S. aureus* than toward *E. coli*, though all sizes are toxic at ≥10^−5^ M to both organisms. We observed a significant difference in size-dependence of antimicrobial activity, which differed based on the microorganism tested. Our work shows that Pd NPs are highly antimicrobial, and that fine-scale (<1 nm) differences in size can alter antimicrobial activity.

## Introduction

The unique chemical and physical properties of nanoparticles (NPs) relative to their bulk counterparts continues to be the driving force behind the discovery of novel applications in several technological areas. Nanoparticles are classified as particles with one dimension within the 1–100 nm size range and behave as a single unit with respect to transport and reactivity. [1] It has been demonstrated that NPs have a wide variety of unique applications, including cell targeting, [2], [3] intravenous nucleic acid delivery, [4]–[6] environmental remediation, [7]–[9] catalysis, [10]–[12] and bactericidal effects. [13]–[15] In particular, silver (Ag) NPs are of pharmaceutical interest because of their potential to replace more traditional synthetic antimicrobial drugs and avoid unintended selection for drug-resistant pathogenic bacterial strains. [16] Multi-drug resistant pathogenic outbreaks are one of the most important global health issues we face today (e.g. extensively drug-resistant tuberculosis, [17] multi-drug resistant cholera [18] and methicillin-resistant *Staphylococcus aureus* [19]) yet have also led to a decrease in investment by pharmaceutical companies into antimicrobial drug development efforts to avoid economic losses when drug-resistant strains develop. [20], [21] As NP research continues, this class of chemicals may provide unique possibilities to address this complex issue. However, as new NP applications continue to emerge, the amount of NPs present in consumer products will inevitably rise, likely leading to large quantities of NPs interacting with humans and the natural environment. [22] In order to conscientiously increase the use of NPs to benefit technological and medical advances, it is essential to understand their implications for human safety and ecosystem health [23]–[25].

While some NPs have been found to have antimicrobial properties (i.e. Ag NPs), a first step toward understanding the broader implications of the impact of novel NPs for any application is to characterize how novel NPs interact with simple biological organisms, such as bacteria. [25]–[38] Studies with single-celled microorganisms can provide a framework for examining relative toxicity of NPs to more complex organisms, multi-trophic level interactions and within complex environmental conditions. They can also provide insight as to whether novel NPs could be useful antimicrobial substances that warrant further study for drug development.

In the present study, we report the first investigation of the activity of well-defined size-controlled palladium (Pd) NPs against bacterial growth. Palladium is one of the most widely used transition metals for carbon–carbon and carbon–heteroatom cross-coupling reactions [39] such as the Suzuki-Miyaura reaction, [40] Heck reaction, [41]–[45] Kumada reaction, [46] Sonogashira reaction, [47] Negishi reaction, [48] Stille reaction, [49] Buchwald-Hartwig reaction, [50] and hydrogenation reactions. [51] Palladium has been used as a catalyst to manufacture pharmaceuticals, [39] degrade harmful environmental pollutants, [52] and as sensors for the detection of various analytes. [53]–[56] Additionally, Pd and Pd^2+^ ions also play a fundamental role in several biotechnological processes. [56] For example, Baccar et al. developed a non-enzymatic biosensor using various sizes of Pd NPs to detect hydrogen peroxide in milk. [56] While the uses of Pd are extensive, advances are yet to be uncovered as the metals are reduced to the nanoscale. Therefore, it is important to determine a baseline of toxicity for Pd NPs as well as examine their potential for antimicrobial applications.

Here, we examine the inhibitory effects of Pd NPs toward gram negative *E. coli* and gram positive *S. aureus* over a 24 h test period. We synthesized three sizes of NPs, each with a narrow size distribution: 2.0±0.1 nm, 2.5±0.2 nm and 3.1±0.2 nm. This work is unique because it examines the effect of NPs synthesized within a very narrow size distribution and it also distinguishes the effects of NPs that vary within a size rage of 0.5 nm. We investigated the influence of Pd NP size, and examined the stability of these NPs throughout the toxicity tests. We also compared the toxicity of Pd NPs relative to Pd^2+^ ions since in some cases Pd^2+^ ions are being replaced by Pd NPs for catalytic applications. This data provide the first assessment of size-dependent antimicrobial effects of novel Pd NPs.

## Materials and Methods

### Materials for Nanoparticle Preparation

Palladium acetate [Pd_3_(OAc)_6_] (OAc = acetate) was purchased from Strem Chemicals (Newbury Port, MA). Dodecyl sulfide was purchased from the Sigma Aldrich Company (Minneapolis, MN). Absolute ethanol (200 proof – ACS grade) was purchased from PHARMCO-AAPER (Shelbyville, KY). We purchased all chemicals and used them as received, without further purification.

### Instrumentation

#### Transmission Electron Microscopy (TEM)

The particle size and size distribution of the Pd nanoparticles in suspension were examined using a Model JEM-1230 JEOL TEM. We dried a 1 µL aliquot of Pd nanoparticles on a 400 mesh Formvar-coated copper grid and placed it in a vacuum desiccator for 5 h.

#### X-ray Diffraction (XRD)

Powder x-ray diffraction (XRD) data were collected using a Scintag XDS Model 2000 diffractometer to analyze the crystalline properties of the Pd NPs. For XRD measurements, we dried and mixed Pd NP samples with 325 mesh Si powder and placed them on a Si wafer sample holder.

#### High-resolution Transmission Electron Microscopy (HRTEM)

We obtained high resolution TEM images and selected area electron diffraction patterns of the Pd NPs using a JEOL 3011 HRTEM at the Center for Microscopy at Michigan State University (East Lansing, MI).

### Synthesis of Pd Nanoparticles (NP) from [Pd_3_(OAc)_6_] by Pyrolysis

To synthesize Pd NPs, we added Pd_3_(OAc)_6_ (0.05 g, 0.075 mmol) and *n*-dodecyl sulfide (0.14 g, 0.37 mmols) to 30 mL of ethanol. We heated the reaction mixture at 90°C for 3 h. We carried out similar reactions in ethanol at 90°C for 1 h and 2 h. The color of the solution turned from orange to dark brown. We removed a 1 µL aliquot of the resulting solutions for TEM imaging. Next, we diluted 10 mL of each of the reaction solutions with 100 mL of milli-Q water (18 MΩ·cm^−1^). We removed a 1 µL aliquot of the diluted solutions for TEM imaging as well. We prepared a solution of 2.5×10^−4^ M Pd^2+^ ions by dissolving a known amount of Pd_3_(OAc)_6_ (0.0084 g, 0.25 mmol L^−1^) in 50 mL of milli-Q water as a control.

### Inhibitory Effects of Pd NPs to Bacteria

Stock cultures of *Escherichia coli* (ATCC strain number 11303) and *Staphylococcus aureus* (ATCC strain number 12600) were obtained from Dr. Silvia Rossbach, Western Michigan University. *E. coli* is a rod-shaped, flagellated microorganism with a gram negative cell wall; *S. aureus* is a coccoid-shaped microorganism with a gram positive cell wall. We grew cultures of *E. coli* and *S. aureus* on sterile Tryptic Soy (TS, Fisher Scientific) plates for 24 h at 37°C. We removed a single colony of each microorganism from a plate and re-cultured it in 10 mL of sterile Bacto Tryptic Soy broth (Fisher Scientific) under the same growth conditions with constant agitation at 200 rpm in a shaking incubator. We then diluted pure cultures from the broth in 200 mL of sterile deionized water to an optical density of 0.889 for *E. coli* and 0.795 for *S. aureus*. We used a BioPhotometer (Eppendorf) to determine the optical density at 600 nm wavelength.

The Pd NPs were prepared and characterized within 24 h of the start of each antimicrobial test as described above. Within 2 h of the start of each antimicrobial test, we prepared a series dilution for each of the different sizes of Pd NPs. The starting concentration of all Pd NPs and Pd^2+^ ions was 2.5×10^−4^ M. The dilution series we used to test for antimicrobial activity included the 2.5×10^−4^ M stock, a 1∶10 dilution of the stock in sterile deionized water, and a series of five 1∶10 dilutions in sterile deionized water. The lowest concentration we tested was 2.5×10^−9^ M. All dilutions were prepared under a sterile hood. We prepared 10 mL of each dilution concentration, plus 10 mL of sterile deionized water as a control; all dilutions and treatments were performed in triplicate. To begin the antimicrobial test, we added 1 mL of the diluted bacterial culture to each dilution tube. Immediately after adding the culture to the dilution tube we vortexed the tube, and plated 75 µL of the treatment onto a TS agar using a spread plating technique. This was the initial (0 h) time point of the experiment. We kept the experimental tubes at 25°C during the course of the 24 h experiment. We repeated the spread plate procedure at 4 h, 8 h and 24 h after initial exposure of the bacteria to the test chemical. We incubated the plates at 37°C for 20 h to grow the bacterial cultures and used a standard darkfield Quebec colony counter (Fisher) to manually count the number of colony forming units (CFU) present on each plate after growth. We chose the absorbance and dilution levels for the two microorganisms based on preliminary data indicating that this procedure would result in comparable and countable colonies (250–300 per plate) when 75 µL of the final dilution was grown using a spread plate procedure. Of the 1440 plates used in this experiment, we classified 48 plates as “uncountable” due to colony spreading. These plates were not included in our statistical analyses of the results. We observed consistent growth in the positive controls for both assays, indicating that comparisons of percent mortality over time were experimentally appropriate. For the *S. aureus* tests, the average CFUs growing in the deionized water controls was 256±10 at 0 h, 222±5 at 4 h, 231±8 at 8 h and 205±9 at 24 h. For the *E. coli* tests, the average colony forming units growing in the deionized water controls was 286±4 at 0 h, 264±3 at 4 h, 256±9 at 8 h and 217±16 at 24 h.

### Statistical Analyses

We conducted Repeated Measures ANOVAs, One-Way ANOVAs and Tukey’s HSD Pairwise Comparisons using Systat Version 13 (SPSS, Inc.) and evaluated significance at α = 0.05. We determined significant differences in colony growth from the control (as presented in the text) and significant differences among the four test chemicals.

## Results

### Nanoparticle Fabrication, Size Control, and Characterization

**Figure 1:**
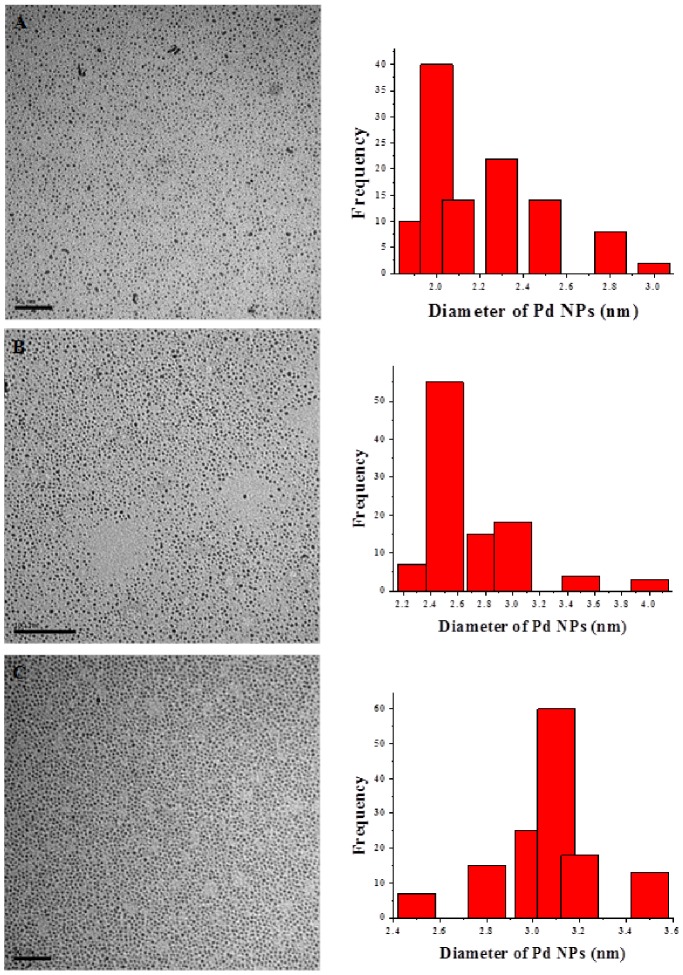
Palladium nanoparticles with a size distribution of 2.0±0.2 nm (A), 2.5±0.1 nm (B) and 3.1±0.1 nm (C).

We prepared Pd NPs from Pd_3_(OAc)_6_ in the presence of *n*-dodecyl sulfide (M:L = 1∶5) in ethanol by a modified pyrolysis reaction. [57] The NP sizes were controlled by varying the length of time of the reaction. After 15 minutes of heating, the reaction solution (for all reactions: 1 h, 2 h, and 3 h) changed color from orange to dark brown, indicating NP formation. We placed a 10 mL aliquot of each reaction mixture in separate 125 mL Erlenmeyer flasks and diluted it with 100 mL of milli-Q water. We removed a 1 µL aliquot of each reaction mixture to prepare a TEM grid. Based on Pd content the concentration of the solutions was 2.5×10^−4^ M. We imaged the NPs to characterize the size and distribution using TEM. Figure 1 shows TEM images of the as-synthesized Pd NPs and their corresponding size histograms. The images show a homogeneous dispersion of the Pd NPs with an adopted spherical shape indicating isotropic growth. We determined the following sizes of the NPs from the TEM images: (a) 2.0 nm, (b) 2.5 nm, (c) 3.1 nm. The TEM images also depict non-aggregated Pd NPs with well controlled sizes.

**Figure 2:**
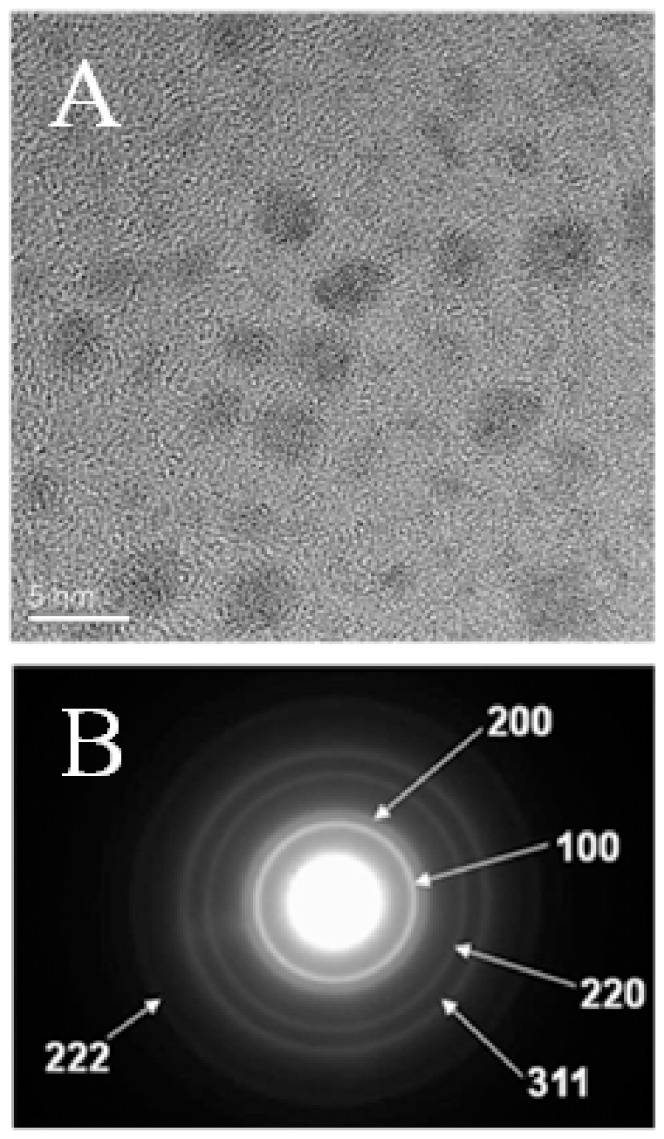
HRTEM image (A) and SAED (B) of *n*-dodecyl sulfide stabilized Pd nanoparticles with size of 3.1±0.1 nm.

**Figure 3:**
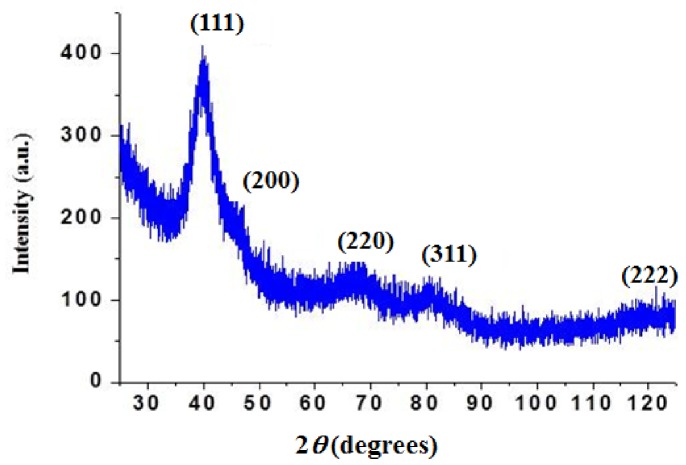
XRD of *n*-dodecyl sulfide stabilized Pd nanoparticles with size of 3.1±0.1 nm.

The HRTEM image (Figure 2a) shows the twinning feature which is typical for most metal nanoparticles. The selected area electron diffraction (SAED) pattern shown in Figure 2b of a single Pd crystal exhibits 5 diffused rings which are assigned to the (111), (200), (220), (311) and (222) reflections that are characteristic of a face-centered cubic (fcc) structure. [57] The powder x-ray diffraction (XRD) (Figure 3) results of the as-synthesized Pd NPs exhibited (111), (200), (220), and (311) diffraction peaks at 40°, 47°, 68°, 82° and120° respectively, and were consistent with the fcc structure of Pd. We calculated the average particle size based on the line broadening of (111) by using the Scherrer formula. Our calculations show that the size is 2.5 nm, which is consistent with the value of the particle measured by TEM (Figure 1), indicating that TEM provides an accurate assessment of Pd NP size.

**Figure 4:**
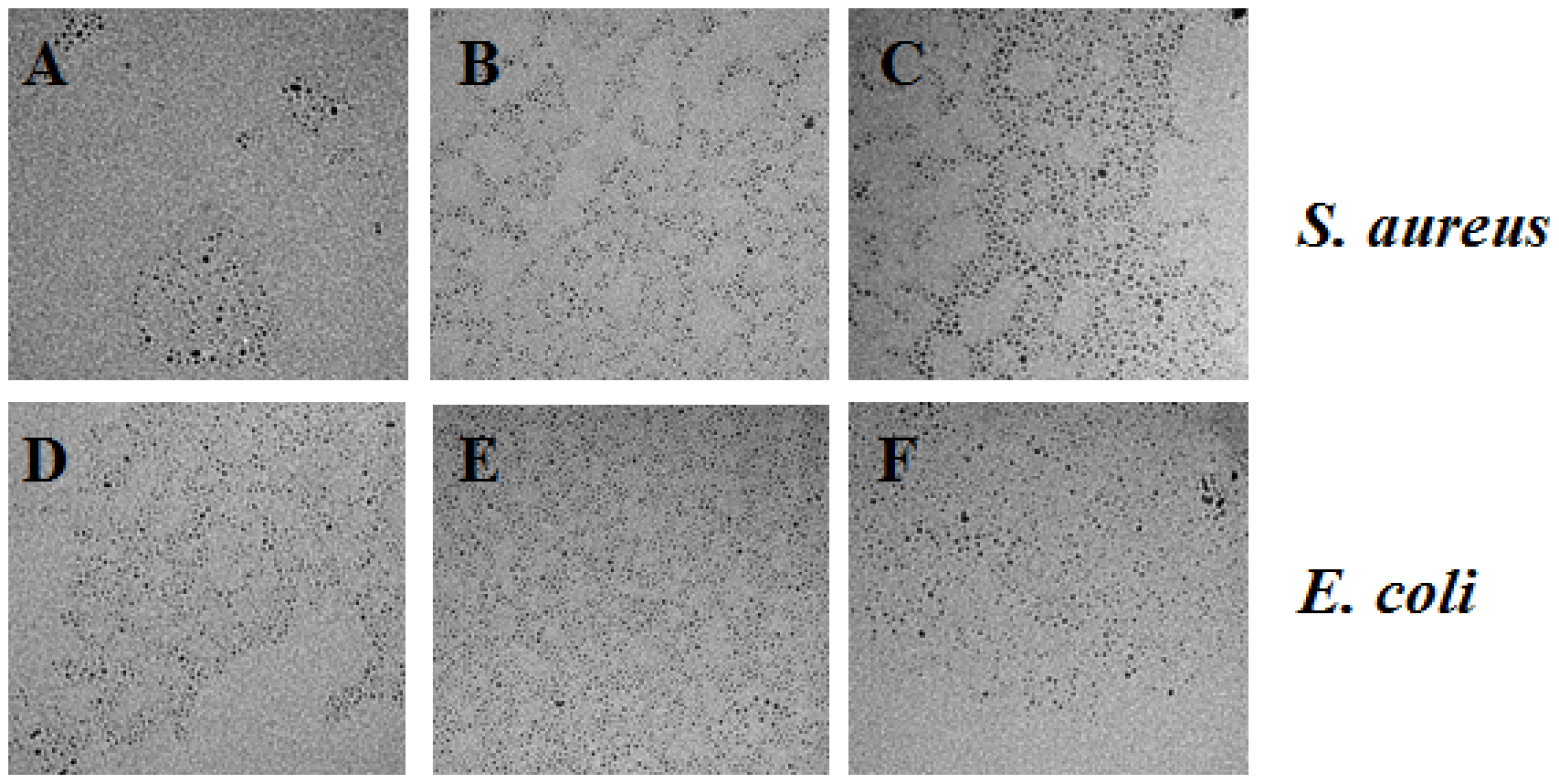
TEM images of the 2.0*S. aureus* and *E. coli* bacteria at the 10^−4^, 10^−5^, 10^−6^ M concentrations. *S. aureus* with 2.0 nm Pd NPs at 10^−4^, 10^−5^, and 10^−6^ M, respectively (A–C). *E. coli* with 2.0 nm Pd NPs at 10^−4^, 10^−5^, and 10^−6^ M, respectively (D–F). Scale bar represents 50 nm.

**Figure 5:**
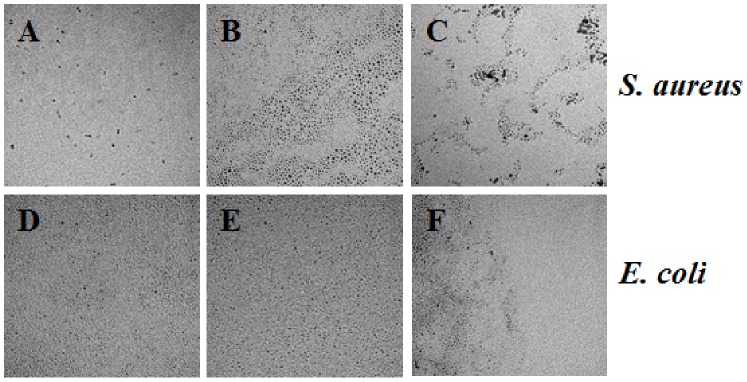
TEM images of the 2.5*S. aureus* and *E. coli* bacteria at the 10^−4^, 10^−5^, 10^−6^ M concentrations. *S. aureus* with 2.5 nm Pd NPs at 10^−4^, 10^−5^, and 10^−6^ M, respectively (A–C). *E. coli* with 2.5 nm Pd NPs at 10^−4^, 10^−5^, and 10^−6^ M, respectively (D–F). Scale bar represents 50 nm.

**Figure 6:**
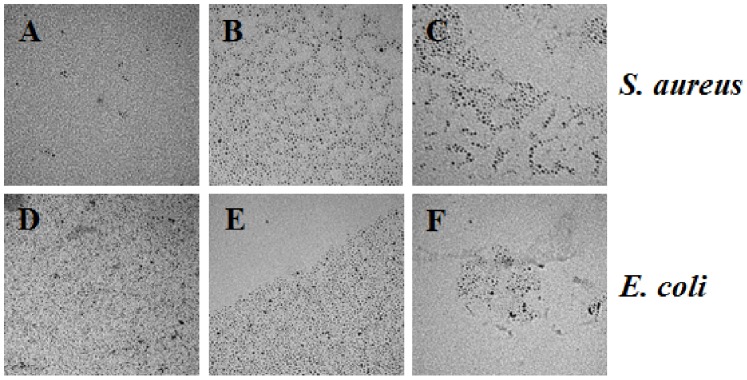
TEM images of the 3.1*S. aureus* and *E. coli* bacteria at the 10^−4^, 10^−5^, 10^−6^ M concentrations. *S. aureus* with 3.1 nm Pd NPs at 10^−4^, 10^−5^, and 10^−6^ M, respectively. (A–C) *E. coli* with 3.1 nm Pd NPs at 10^−4^, 10^−5^, and 10^−6^ M, respectively (D–F). Scale bar represents 50 nm.

We captured additional TEM images to depict the exposure of Pd NPs toward *S. aureus* and *E. coli* at all concentrations (2.5 ×10^−4^, 10^−5^, 10^−6^, 10^−7^, 10^−8^, and 10^−9^ M) for each size of nanomaterials after 24 h. Figures 4–6 show the NP exposure to bacteria for the three higher concentrations (2.5×10^−4^, 10^−5^, 10^−6^ M). It is illustrated that no significant change in size and/or shape can be observed after the Pd NPs have been exposed to the bacteria. Throughout the remainder of the paper, we will refer to the molar concentrations of Pd NPs by the order of magnitude only.

### Toxicity to Two Bacterial Populations Immediately after Exposure to Pd NPs

**Figure 7:**
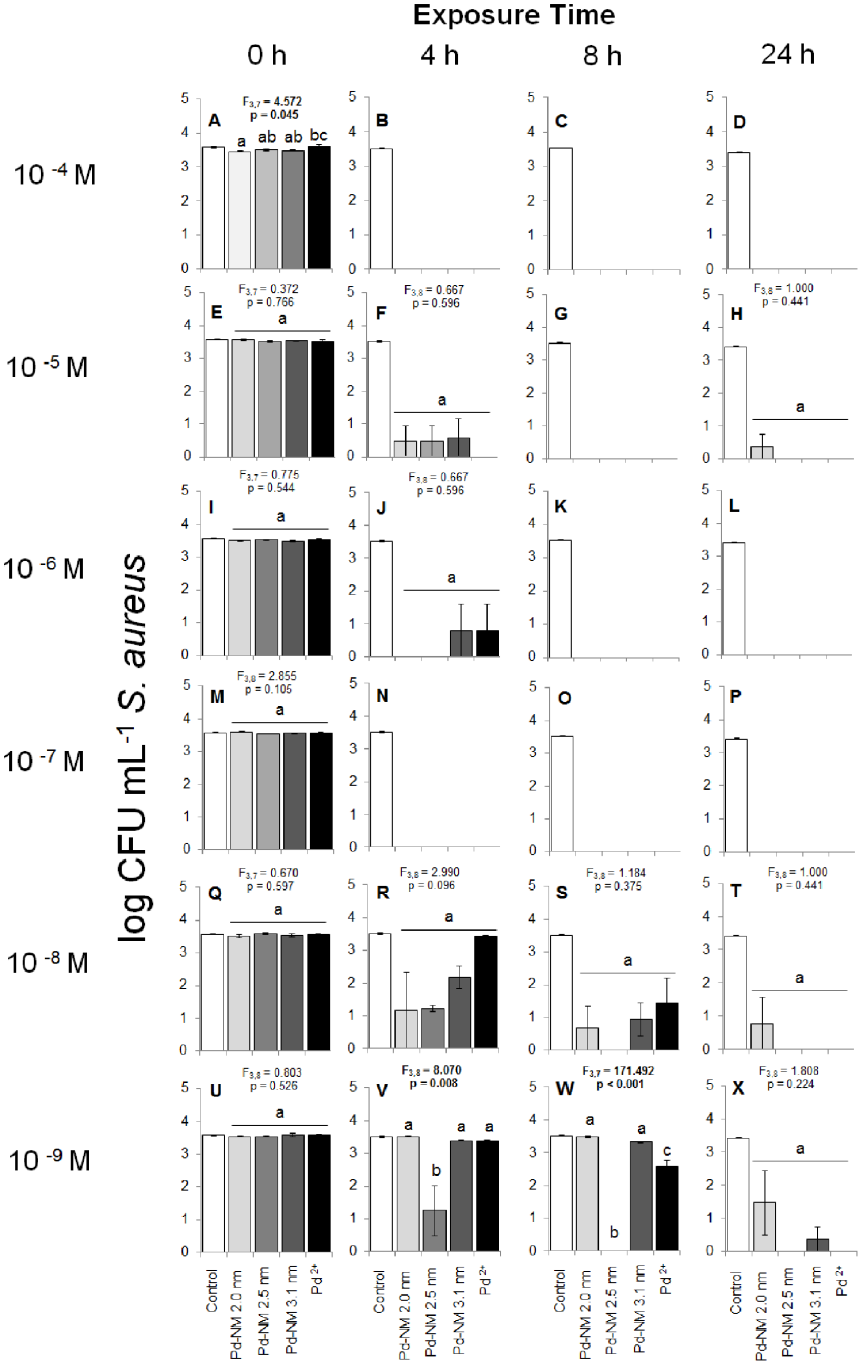
Colony forming units mL^−1^ of *S. aureus* on TS agar plates following exposure to a sterile deionized water control, and treatments of 2.0, 2.5 and 3.1 nm-sized Pd NPs and Pd^2+^ ions. Exposure times are within 5 minutes of exposure (0 h), and after 4 h, 8 h and 24 h (horizontal). The concentration range of each of the Pd NPs and Pd^2+^ tests were from 10^−4^–10^−9^ M (vertical). Significant differences shown in the figure represent comparisons between the four test chemicals using One-Way ANOVA and do not include comparison to the control (white bar); significance is represented by lowercase letters. Significance was assessed at α = 0.05. Capitalized letters refer to individual sub-figures A–X, as corresponds to exposure time and concentration.

**Figure 8:**
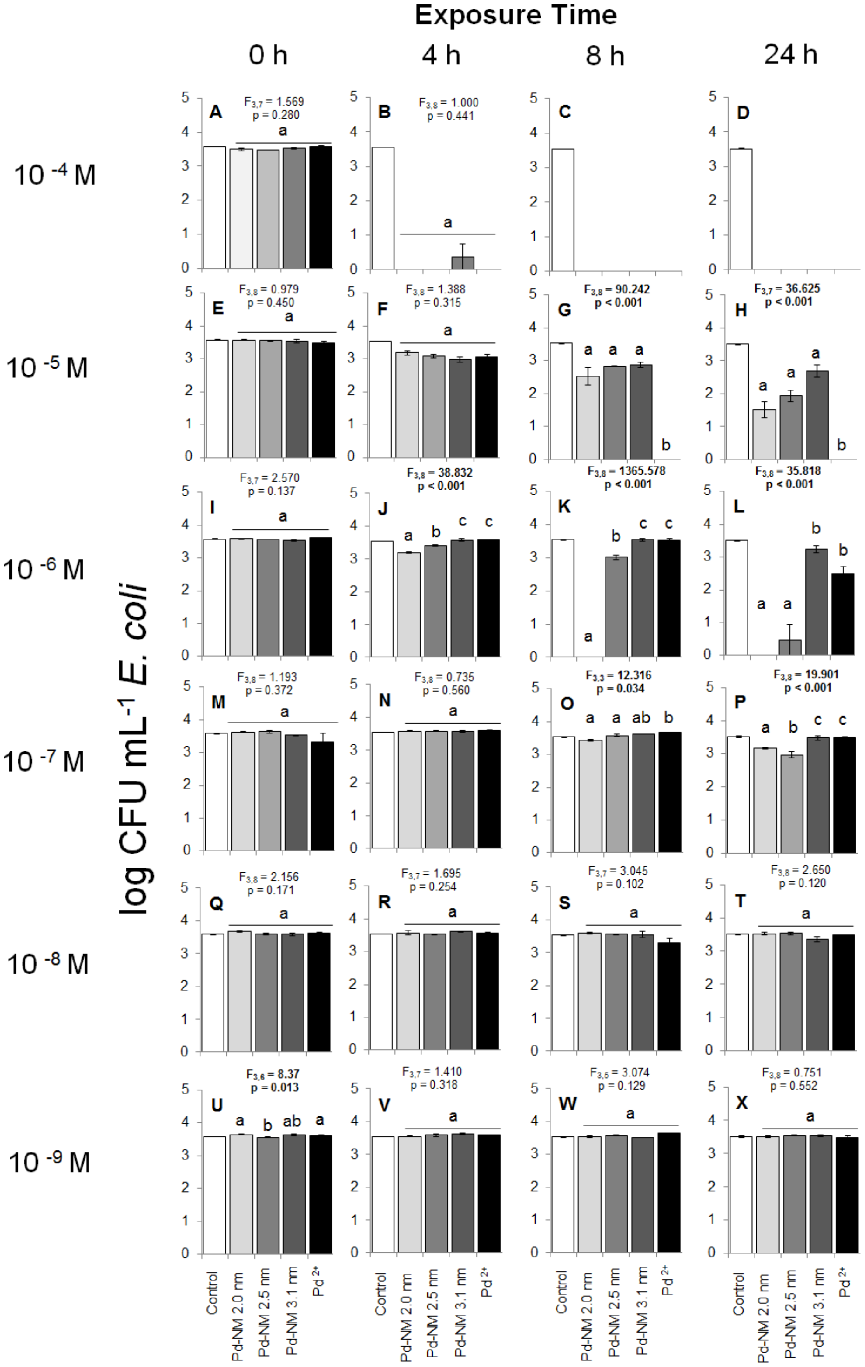
Colony forming units mL^−1^ of *E.coli* on TS agar plates following exposure to a sterile deionized water control, and treatments of 2.0, 2.5 and 3.1 nm-sized Pd NPs and Pd^2+^ ions. Exposure times are within 5 minutes of exposure (0 h), and after 4 h, 8 h and 24 h (horizontal). The concentration range of each of the Pd NPs and Pd^2+^ tests were from 10^−4^–10^−9^ M (vertical). Significant differences shown in the figure represent comparisons between the four test chemicals using One-Way ANOVA and do not include comparison to the control (white bar); significance is represented by lowercase letters. Significance was assessed at α = 0.05. Capitalized letters refer to individual sub-figures A–X, as corresponds to exposure time and concentration.

We examined the inhibitory effects of six concentrations for each of the different sized (2.0, 2.5, and 3.1 nm) Pd NPs and Pd^2+^ ions to the growth of pure cultures of *E. coli* and *S. aureus* over a 24 h period. At the initial time point (0 h), we observed only minor differences in the amount of colony forming units (CFU) mL^−1^ between any treatment and the control (Figures 7A, 7E, 7I, 7M, 7Q, 7U & Figures 8A, 8E, 8I, 8M, 8Q, 8U). In the *S. aureus* tests, all Pd NP treatments resulted in the same amount of *S. aureus* colony growth mL^−1^ as the control, with the exception of 10^−4^ M test (Figure 7A), where the 2.0 nm Pd NP test resulted in marginally fewer colonies than the control (Tukey’s HSD, p = 0.047). In the *E. coli* tests, all Pd NP treatments resulted in the same amount of *E. coli* CFU mL^−1^ as the control, with the exception of the 10^−4^ M and 10^−9^ M concentration tests. In the 10^−4^ M test with *E. coli* (Figure 8A), fewer colonies grew in the 2.5 nm-sized Pd NP treatment as compared to the control (Tukey’s HSD, p = 0.018). In the 10^−9^ M test with *E. coli* (Figure 8U), a slightly larger number of colonies grew in the 2.0 nm Pd NP test as compared to the control (Tukey’s HSD, p = 0.025). In general, differences at 0 h between the four Pd NP treatments and the control were minor, indicating no immediate effects of the Pd NPs on the growth of either bacterium.

### Toxicity of Pd NPs to Two Bacterial Populations Over Time

For both microorganisms, we observed a significant decrease, as compared to the control, in the amount of colonies mL^−1^ over the 24 h-exposure time for all four test chemicals at the 10^−4^, 10^−5^ and 10^−6^ M concentrations (repeated measures ANOVA, p<0.001). At the 10^−4^ M concentration, all four test chemicals induced a near-100% mortality rate in both microorganisms after 4 h of exposure (Figures 7B & 8B). We observed growth of *E. coli* colonies in the 10^−4^ M 3.1 nm Pd NP treatment at 4 h (Figure 8B), but average colony growth did not differ significantly from 0 CFU mL^−1^ (F_3,8_ = 1.000, p = 0.441). At 8 h and 24 h, the 10^−4^ M concentration of all four test chemicals completely inhibited growth of both bacteria (Figures 7C, 7D & Figures 8C, 8D). At 10^−5^ M concentration, all four test chemicals induced near 100% mortality in the *S. aureus* tests, as compared to the control (p<0.001; Figures 7F, 7G, 7H). We observed growth of *S. aureus* colonies in all three Pd NP treatments at 4 h (Figure 7F) and in the 2.0 nm Pd NP test at 24 h (Figure 7H). However, the average colony growth did not differ significantly from 0 CFU mL^−1^. Similarly, 10^−5^ M concentrations of all four test chemicals were inhibitory toward *E. coli* at 4 h, 8 h and 24 h as compared to the control (p<0.001; Figures 8F, 8G, 8H). However, the magnitude of inhibition by the three Pd NPs at 10^−5^ M concentrations was far less in the *E. coli* tests than in the *S. aureus* tests. For example, after 4 h of exposure, the 2.0 nm and 2.5 nm Pd NMs were 85% more inhibitory to *S. aureus* than to *E. coli*, the 3.1 nm Pd NM was 81% more inhibitory and the Pd^2+^ ions were 100% more inhibitory (Figure 7F & Figure 8F). By 8 h and 24 h, all three Pd NMs remained less inhibitory to *E. coli* than to *S. aureus*, but the Pd^2+^ ions completely inhibited growth of both bacteria (Figure 7G, 7H & Figure 8G, 8H).

We continued to observe a trend of nearly complete inhibition by all four test chemicals to *S. aureus* colonies in the 10^−6^ M and 10^−7^ M tests beyond 4 h of exposure (Figures 7J, 7K, 7L, 7N, 7O, 7P). *S. aureus* grew in the 10^−6^ M test in the 3.1 nm Pd NP and Pd ^2+^ tests (Figure 7J), but again, the amount of growth did not differ significantly from 0 CFU mL^−1^. Conversely, at 10^−6^ M concentrations at 4 h and 8 h of exposure, only the 2.0 nm and 2.5 nm Pd NPs were significantly inhibitory toward *E. coli*, as compared to the control (Tukey’s HSD, p<0.001 and p = 0.025 respectively at 4h; p<0.001 at 8 h in both cases). The 3.1 nm Pd NPs and Pd^2+^ test results did not differ from the control at 4 h and 8 h (Figures 8J, 8K). By 24 h of exposure at 10^−6^ M concentration, all four test chemicals significantly inhibited *E. coli* growth (Figure 8L), though we observed nearly complete inhibition by the 2.0 nm and 2.5 nm Pd NPs, and only 7% inhibition by the 3.1 nm Pd NP and 28% inhibition by the Pd^2+^ ions (Figure 8L).

The first sub-lethal effects we saw of the test chemicals toward *S. aureus* were in the 10^−8^ M concentration test at 4 h (Figure 7R). Statistically, all four chemicals tested were equivalently inhibitory toward *S. aureus* (F_3,8_ = 2.990, p = 0.096). As exposure time continued, we observed a similar trend of inhibition toward *S. aureus* at 8 h and 24 h in the 10^−8^ M concentration tests (Figures 7S, 7T). Though some growth did occur at 8 h and 24 h, average colony growth in these treatments did not differ significantly from 0 CFU mL^−1^. Conversely, none of the treatments at 10^−8^ M concentrations significantly reduced the growth of *E. coli*, as compared to the control (One-way ANOVA: F_4,9_ at 4 h = 1.838, p = 0.206, F_4,9_ at 8 h = 2.953, p = 0.082, F_4,10_ at 24 h = 2.363, p = 0.123; Figures 8R, 8S, 8T).

The trend of no difference in toxicity toward *E. coli* between any treatment and the control continued in the 10^−9^ M concentration tests at all time points (One-way ANOVA: F_4,9_ at 4 h = 2.118, p = 0.161, F_4,7_ at 8 h = 3.368, p = 0.077, F_4,10_ at 24 h = 0.562, p = 0.696; Figures 8V, 8W, 8X). Similarly, after 4 h of exposure the 2.0 nm, 3.1 nm and Pd^2+^ ion treatments did not impact the growth of *S. aureus* in the 10^−9^ M concentration tests, as compared to the control (Figure 7V). However, the 2.5 nm Pd NP significantly inhibited *S. aureus* growth after 4 h of exposure, as compared to the control (Tukey’s HSD: p = 0.006). By 8 h of exposure in the 10^−9^ M concentration tests, both the 2.5 nm and 3.1 nm Pd NPs significantly inhibited the growth of *S. aureus* (Tukey’s HSD: p<0.001, Figure 7W), but the 2.0 nm Pd NP and the Pd^2+^ ions did not reduce *S. aureus* growth, as compared to the control. After 24 h of exposure, all four test chemicals inhibited *S. aureus* growth significantly (Figure 7X). While we did observe *S. aureus* colonies in the 2.0 nm and 3.1 nm Pd NP tests at 24 h, the average growth in these treatments did not differ significantly from 0 CFU mL^−1^. Thus, extremely low (10^−9^ M concentrations) of all Pd NPs and Pd^2+^ ions inhibited growth of *S. aureus* after 24 h of exposure (Figure 7X), but did not inhibit growth of *E. coli* (Figure 8X).

### Size-Dependence of Toxicity

Given the high levels of growth inhibition toward *S. aureus*, we only observed variances in the effects of the different-sized NPs in the 10^−9^ M concentration test at the 4 h and 8 h time points (Figures 7V, 7W). At 4 h, the 2.5 nm-sized Pd NPs were more inhibitory to *S. aureus* colony growth than any of the other test chemicals (F_3,8_ = 8.070, p<0.008, Figure 7V). The effects of 10^−9^ M concentrations of 2.0 nm, 3.1 nm Pd NPs and the Pd^2+^ ions did not differ at the 4 h time point. These results indicate that the mid-sized (2.5 nm) particles are the most toxic to *S. aureus*, while smaller (2.0 nm) and larger (3.1 nm) NPs and Pd^2+^ ions are less toxic. This basic trend was also observed in the 10^−9^ M test at 8 h (Figure 7W) and in the 10^−8^ M test at 4 h and 8 h (Figures 7R, 7S), though it was not a significant effect in the 10^−8^ M tests. By 24 h of exposure, we observed no difference in the effect of any of the NP sizes on *S. aureus* growth, indicating that the size-dependent toxicity effect is also time-dependent.

The trend in size-dependent toxicity differed between the *S. aureus* and *E. coli* tests. In the *E. coli* test, we observed differences in the effects of the four test chemicals most obviously in the 10^−6^ M concentration test (Figures 8J, 8K, 8L). At 4 h, 8 h and 24 h exposure times at this test concentration, the smallest 2.0 nm-sized Pd NP exhibited the greatest inhibitory effects toward *E. coli* (F_3,8_ = 38.832, p<0.001). The 2.5 nm Pd NPs were the second most inhibitory toward *E. coli* at all time points, followed by the largest 3.1 nm Pd NPs, which were the least inhibitory. The effects of the Pd^2+^ ions on *E. coli* colony formation did not differ from the 3.1 nm Pd NPs at 4 h and 8 h, but were equally inhibitory to the 2.0 nm and 2.5 nm NPs at 24 h. This basic trend that the smaller Pd NPs were more toxic to *E. coli* than the largest NP was also observed in the 10^−7^ M concentration test, particularly at 24 h (Figures 8P).

## Discussion

While many previous studies have successfully demonstrated the toxic effects of different size ranges of NPs on bacteria, most have not been able to test the for the potential effects of NPs with narrow size distributions (i.e. ±0.2 nm) and these studies do not distinguish between particle sizes that vary <1 nm size changes on bacterial toxicity. This is primarily because the commercially available particles used in most studies lack monodispersity. Our ability to control the size of the NPs we synthesize and obtain them with narrow size distributions gives the added advantage that we can determine how specific sizes (i.e. ±0.2 nm) affect bacterial toxicity. In general, traditional toxicological studies to determine lethal and effective doses using NPs as test chemicals are highly challenging, because nanomaterial size and shape is often inconsistent from test to test and manufactured NPs consist of a range of sizes. For example, commercially purchased TiO_2_ NPs that were described as having a particle size of 5 nm by the manufacturer were shown to have an actual particle size of 3.5±1.0 nm (size range of 2–5 nm). [58] Similarly, manufactured Al NPs, Al_2_O_3_ NPs and Al_2_O_3_ nanowhiskers purchased from a supplier were shown to be highly polydispersed and batch-dependent. [59] Thus, it was crucial for us to perform toxicity assays to new NPs only with well-characterized test particles.

In this study, we examined how <1 nm differences in Pd NP sizes influence microbial growth to determine if Pd NPs are viable antimicrobial materials for future study and if their antimicrobial activity can be fine-tuned to target specific types of microorganisms. The as-synthesized Pd NPs used in our study were monodisperse with a narrow size distribution (2.0±0.1 nm, 2.5±0.2 nm, 3.1±0.2 nm). This unique feature is produced by controlling the size and shape through an effective synthetic pathway. The NP size was directly proportional to the reaction time. Thus, as the reaction time increased so did the particle size which is consistent with the Oswald ripening mechanism; as the reflux or heating time is prolonged the NP diameter increases. [60] The size of the NP can easily be tuned by varying the ratio of the reactants and or reaction conditions (metal precursor, stabilizer, reducing agent, time, temperature). [57] Aggregation has been shown to play a role in reducing the surface area of the particle which ultimately affects the surface properties. [61]–[63] In turn, NP aggregation can result in dissolution whereby metal atoms or ions are generated that may have toxic effects. [64] The small size of the Pd NPs is an important aspect of this study because previous work has shown that the electronic structure of the surface of the nanoparticles will change as a function of the size, and ultimately enhance the surface reactivity. [65], [66] In particular, nanoparticles with sizes <10 nm can attach to the surface of the cell membrane of the bacteria and disrupt the normal function (e.g. respiration and permeability). [65].

Many types of NPs have been preliminarily examined using bacterial test systems, and show significant toxic effects toward bacteria. These include metallic particles (Ag, Fe),[26]–[29], [65], [67]–[71] semiconductors (ZnO, TiO_2_, Al_2_O_3_, SiO_2_, MgO), [30]–[36], [72]–[74] quantum dots (CdTe, CdSe), [75] nano-carbon (SWCNTs/MWCNTs, C60), [76], [77] and rare earth nanoparticles (CeO_2_, La_2_O_3_). [37], [38] To our knowledge, Pd NPs have not been examined for their antimicrobial activity previously. Among the inorganic metallic NPs that are most similar to the Pd NPs used in this study, Ag NPs have been most highly examined. They have been shown to exhibit significant toxic effects toward several bacterial, [13]–[15], [26]–[28], [65], [78] fungal, [79] algal [80] and mammalian cell lines. [81] Results indicate that Ag NPs are significantly bactericidal, accumulate in bacterial cell membranes, [28] and impact bacterial respiratory and cell signaling pathways. [15], [27], [82], [83] Additionally, smaller particles have been shown to have a greater inhibitory effect on bacterial growth than larger particles. [65], [84] This trend was exhibited in our study with Pd-based NPs in the *E. coli* toxicity tests. Conversely, the smallest particles were least toxic to *S. aureus* at the first sub-lethal concentration (10^−9 ^M), while the mid-sized 2.5 nm Pd NPs were most toxic. Several studies have reported on the comparison of NP (Ag, MgO, ZnO, TiO_2_, SiO_2_, Al_2_O_3_, La_2_O_3_) toxicity to bacteria with gram negative versus gram positive cell walls. [27], [33], [37], [69], [73] In general, Ag NPs inhibit growth of gram negative *E. coli* (strain O157:H8) at lower concentrations than they inhibit gram positive *S. aureus*. [14] Our results overwhelmingly show the opposite trend: that Pd NPs are highly antimicrobial, and that they are more inhibitory to gram positive *S. aureus* than to gram negative *E. coli*. An extremely low concentration (10^−9 ^M) of all test chemicals induced a significant inhibitory effect to *S. aureus* after 24 h exposure. In contrast, none of the NPs tested were toxic to *E. coli* below a concentration of 10^−6^ M.

The mechanism by which Pd NPs are more toxic to *S. aureus* than to *E. coli* or why the 2.5 nm Pd NP was most toxic to *S. aureus* at sub-lethal concentrations is not clear from our current work and warrants further study. One likely explanation for these results is that the size of highly constrained Pd NPs used in our tests ranged from 1–3 nm, whereas studies of polydispersed Ag NPs used a higher size limit of 5–50 nm NPs. [26]–[28], [65], [68]–[70], [85] *S. aureus* cells are 1 µm diameter spheres that have a surface area of approximately 3 µm^2^, whereas *E. coli* cells, which are 1 µm wide by 3 µm long rods, have a surface area of approximately 11 µm^2^. If size is the only factor considered, >10 nm NPs would interact more easily with *E. coli* cells than *S. aureus* cells, simply given size and steric constraints. However, using smaller 2–3 nm NPs used in this study, the reaction sites for Pd on *S. aureus* cells would become saturated much more quickly and at much lower concentrations than *E. coli* cells.

Another possibility is that Pd NPs become ionized during the course of the toxicity assays. It is well known that gram negative bacteria, such as *E. coli*, can utilize efflux complexes to remove toxic compounds from the cell. For example, the CusCBA efflux system is responsible for removing biocidal copper and silver ions from *E. coli* cells. [86] Efflux pumps contribute significantly to the problem of acquired bacterial antibiotic resistance because of the broad variety of substrates they can recognize. [87] While it is possible that the *E. coli* cells in our tests effectively removed Pd NPs after they had entered the cell, our time-series TEM images of Pd NPs (Figures 4–6) suggest that Pd NPs do not change in size and are not oxidized over the test period.

It is most likely that surface area interactions between cell walls and Pd NPs are confounded by cell wall type. The outer membrane of gram negative bacteria, composed of a lipopolysaccharide-phospholipid asymmetric bilayer, bacteria provides a significant barrier to antimicrobial compounds. [88] For example, antibiotics can only penetrate into gram negative cells through lipid-mediated pathways and general diffusion porins. [89] The composition of the outer membrane of gram negative bacteria versus the primarily peptidoglycan-based cell wall of gram positive bacteria can significantly influence the ability of metals to bind to the bacterial surfaces. [90] Gram positive cell walls have been shown to act as metal chelators, whereas different transition elements (II) have been shown to vary widely in binding efficiency to *E. coli* AB264 cell envelopes. [91], [92] For example, Pd metal binds with relatively low efficiency to *E. coli* cell envelopes (0.010 µmol mg^−1^) as compared to iron (III) (0.200 µmol mg^−1^) and cobalt (0.178 µmol mg^−1^). [92] In comparison, little is known about the binding efficiency of metals in the nano form to gram positive and gram negative bacteria. Morones et al. showed that small-scale Ag NPs (5 nm ±2 nm ) interacted directly with bacterial cells by attaching to the membrane and penetrating the cell. [65] This is because Ag NPs create changes in membrane morphology which increase membrane permeability and disrupt proper transport of molecules to the cytoplasm. [28], [65] When examined using TEM following treatment with Ag NPs, *E. coli* cells exhibit significant damage to membranes, in the form of pits on their surfaces. [28].

Surprisingly, the 2.0 nm Pd NPs were less toxic to *E. coli* at 10^−5^ M concentrations than at 10^−6^ M concentrations (Figures 8G, 8K). In both cases, all three of the replicate treatment tubes and plates were used to generate the calculated averages and the error surrounding the averages was small, indicating that these results were not obtained by chance alone. A similar result was seen in the *S. aureus* tests, where 2.0 nm NPs allowed more colony growth at 10^−5^ M than at 10^−6^ M concentrations at the 4 h time point (Figures 7F, 7J). However, in the *S. aureus* test, the error surrounding the average colony growth was large enough that the colony growth we observed at 10^−5^ M at 4 h was not significantly different from 0 CFU mL^−1^. Further investigation is required to determine the mechanism by which Pd NPs are toxic to both *E. coli* and *S. aureus* to explain this result. An obvious explanation of this pattern would be that the NPs formed aggregates at the 10^−5^ M concentration, but did not form aggregates at lower concentrations. However, we tested for agglomeration of the Pd NPs at 10^−5^ M with both bacterial cultures and observed no notable agglomeration occurring (Figures 4 and 6).

While the mode of action of Pd NPs remains unknown, Pd^2+^ activity as an enzyme inhibitor is well studied. Pd^2+^ is known to inhibit creatine kinase, succinate dehydrogenase, and many other common enzymatic processes in both prokaryotic and eukaryotic cells. [93] If entry into the cytoplasm in high quantities instead of membrane disruption is required to elicit Pd toxic effects, then smaller cell size and fundamentally different cell wall properties of gram positive *S. aureus* may render it more susceptible to Pd NPs than larger gram negative cells. By examining the toxicity of well-constrained particle sizes with <1 nm differences in size, we have demonstrated that fine-tuning the sizes of nanomaterials can have beneficial results for targeting particular bacterial cell types. In this case, 2.5 nm Pd NPs are the ideal size to target *S. aureus*, and will cause nearly 100% mortality of an *S. aureus* culture within 4 h of exposure to a mere 10^−9^ M concentration of that particular NP. The results we obtained are closely related to work done with other nanoparticles, however, the size of the NPs we used have a much narrower distribution relative to other types of nanoparticles reported in the literature. [13]–[15], [26], [34] Such particles with narrow size distributions enable is to understand the effect of a specific particle size on a bacterial strain, and also how changing the size by a size as small as 0.5 nm affects the interaction with microorganisms.

Using either test organism, the antimicrobial activity of Pd NPs of all sizes was high at low concentrations, indicating that Pd NPs are useful targets for future study of novel antimicrobial compounds. It is inevitable that Pd NPs will make their way into the market and that increased use and release of Pd NPs into wastewater streams and into the natural environment should be carefully regulated. When studied under more complex test conditions, determining the impacts of NPs becomes far from straightforward. For example, Ag NPs are four times less inhibitory to *E. coli* cells in a biofilm than to planktonic *E. coli* cells, likely due to aggregation of Ag NPs which inhibited diffusion throughout the biofilm. [67] Further studies investigating the mechanism of toxicity of Pd NPs and the effects of all NPs to ecological systems are a crucial target research area for these high functioning novel materials.

### Conclusions

Investigating the interactions of metal NPs with bacterial cells is an important step toward realizing the toxicity of NPs but also in identifying potential biological applications. Pd NPs were found to be more toxic than Pd^2+^ ions when exposed to gram positive *S. aureus* bacteria. Nanoparticles with smaller diameter, i.e. 2.0±0.1 nm were found to exhibit higher toxicity relative to the 2.5±0.2 nm and the 3.1±0.2 nm. The inhibitory growth effect was evident upon contact with the bacteria at concentrations as low as 10^−9^ M of Pd NPs. Relative to gram positive *S. aureus*, the gram negative *E. coli* required higher concentrations of Pd NPs and longer exposure times before an inhibitory growth effect became evident. This observation is apparent that Pd NPs could be useful antimicrobial agents for gram positive bacteria.
